# Self-Regulatory Goal Motivational Processes in Sustained New Year Resolution Pursuit and Mental Wellbeing

**DOI:** 10.3390/ijerph18063084

**Published:** 2021-03-17

**Authors:** Joanne M. Dickson, Nicholas J. Moberly, David Preece, Alyson Dodd, Christopher D. Huntley

**Affiliations:** 1 Psychology Division, School of Arts & Humanities, Edith Cowan University, Joondalup 6027, Australia; 2 Department of Psychology, University of Exeter, Exeter EX4 4QG, UK; n.j.moberly@exeter.ac.uk; 3 School of Population Health, Curtin University, Bentley 6102, Australia; david.preece@curtin.edu.au; 4 Department of Psychology, Faculty of Health and Life Sciences, Northumbria University, Newcastle upon Tyne NE1 8ST, UK; alyson.dodd@northumbria.ac.uk; 5 Faculty of Health and Life Sciences, School of Medicine, University of Liverpool, Liverpool L69 3GB, UK; C.Huntley@liverpool.ac.uk

**Keywords:** wellbeing, New Year resolutions, flexible goal adjustment, goal flexibility, goal tenacity, New Year resolution “stickability”, New Year resolution goal characteristics, self-regulation

## Abstract

Recent research suggests people typically “give up” pursuing their New Year resolutions within the first month. The present study investigated goal features proposed to be implicated in promoting both mental wellbeing and sustained New Year resolution pursuit. Australian and UK participants (*n* = 182) took part in an online longitudinal study, including four timepoints over a two-month period. At baseline, participants listed the New Year resolution to which they were most committed, and completed self-report measures to assess mental wellbeing, goal flexibility and tenacity. At the follow-up surveys, participants completed the wellbeing measure and their New Year resolution commitment, effort and stickability. As predicted, flexibility predicted wellbeing across time, however, tenacity did not. Counter to prediction, neither flexibility nor tenacity reported at baseline predicted “sticking” with one’s New Year resolution. The predicted interaction between flexibility and tenacity was not significant. New Year resolutions focused predominantly on “diet” and “exercise” were predominantly the same resolutions previously pursued and tended to be relatively abstract. Although goal flexibility predicted greater wellbeing, the findings overall tend to support the view that people are not particularly good at sticking with their New Year resolutions. Implications of the findings are discussed.

## 1. Introduction

Personal goal striving is fundamental to human experience and drives much everyday human behaviour. It promotes positive adaptations in life and psychological wellbeing, even when goal pursuit is not successful [1]. Personal goals are defined as cognitive representations of desired future outcomes that involve striving toward the positive outcome [2]. One form of goal pursuit that has received little empirical attention are New Year resolutions. Yet, as each New Year is welcomed in across the globe a common activity many individuals engage in is the setting of New Year resolutions. The New Year offers an opportunity for people to take stock, to reflect on the past year and to set resolutions they wish to pursue in the coming year. As with personal goals, New Year resolutions typically require people to take goal-congruent actions, to sustain pursuit in the face of setbacks and obstacles, whilst resisting the pull of competing goals to achieve the desired outcome(s). The scant research undertaken in this area suggests that people tend not to “stick” to their New Year’s resolutions beyond a few weeks [3]. Relatively little is known about specific goal mechanisms that may sustain New Year resolution striving and promote subjective wellbeing over time. Here, for the first time, we aim to examine two specific New Year resolution-related mechanisms (i.e., goal flexibility and goal tenacity) that may be implicated both in mental wellbeing and the ability to stick to one’s New Year resolutions (i.e., the ability to keep striving towards the resolution outcome).

Goal flexibility refers to the ability to view setbacks with equanimity and adjust goal pursuit as required, whereas goal tenacity is defined as persistence in striving to reach a desired goal outcome under difficult conditions [4]. Goal theorists posit that flexible goal adjustment is integral to maintaining a sense of well-being [5,6]. There is often more than one way to reach a desired outcome when difficulties arise. One may flexibly switch to an alternative means to pursuing the same goal [7], or one may switch to a new goal that serves the same superordinate goal as the original goal [8]. Hence, the ability to be flexible, to adjust to setbacks and to create alternative striving strategies as required should be advantageous for facilitating a sustained pursuit of New Year resolutions and maintaining a sense of wellbeing, which this study aims to investigate. In contrast, inflexible goal processes are thought to give rise to psychological difficulties [9,10], and are apt to hinder an effective New Year resolution pursuit. For instance, research has consistently characterised depression in terms of inflexible conditional goal setting [5,6,11]. Theoretically, inflexible pursuit in the face of an unattainable goal is hypothesised to lead to a downward spiral of negative self-criticism, self-evaluation and depressive symptoms [10].

In sharp contrast to goal flexibility, less research has investigated goal tenacity, which has been defined as persistence [12]. Arguably, persistence is likely to provide the necessary impetus and drive required to sustain New Year resolution pursuit in the face of difficulties and obstacles. While the construct of goal tenacity may overlap with other constructs such as grit, goal tenacity and goal flexibility are focused specifically on coping with setbacks to goal pursuit through action regulation [12]. The interplay between goal tenacity and goal flexibility is also thought to be adaptive for goal pursuit, and this was of particular interest in this study. The few studies that have investigated a tenacious goal pursuit and goal flexibility in relation to wellbeing have used older adult populations. This research suggests that goal flexibility and tenacity each independently predict wellbeing [12,13]. However, no studies have examined a goal tenacity or flexibility in relation to one’s ability to stick to New Year resolutions.

According to Brandtstädter and colleagues (1990), key life transitions activate two distinct but complementary modes of coping with obstacles, which represent “assimilative” (i.e., transforming developmental circumstances in accordance with personal preferences) and “accommodative” (i.e., adjusting personal preferences to situational concerns) tendencies [13]. These “assimilative” and “accommodative” tendencies are measured by two independent goal scales, tenacious goal pursuit (TGP) and flexible goal pursuit (FGP). Both scales have been shown to independently predict high life satisfaction and low depression, and to be positively related to generalised internal control beliefs [13]. A longitudinal cohort study, in an aging population, found that flexible and tenacious goal pursuit each independently predicted subjective wellbeing (SWB), and the interaction between flexibility and tenacity significantly predicted increased positive changes, such that more flexible and tenacious individuals reported the largest decreases in symptoms of depression, hostility and physical ill-health [12,14]. However, decreases in depressive symptoms are not synonymous with mental wellbeing. According to the World Health Organisation (WHO) wellbeing is more than the absence of psychological distress [15].

Here, for the first time, we aim to investigate whether goal flexibility and tenacity each, independently, predict increased wellbeing and sustained New Year resolution goal pursuit (i.e., New Year resolution “stickability”) using Australian and the UK community samples. Next, we aim to investigate whether the interactive effects of goal flexibility and goal tenacity will make an additional contribution to predicting wellbeing and sustained New Year resolution “stickability”.

### Predictions

First, we predicted that goal flexibility and goal tenacity, measured at baseline, would each significantly and independently predict higher levels of mental wellbeing over time. Next, we predicted that the interactive association of goal flexibility and goal tenacity would predict further increases in mental wellbeing over time. In relation to sustained New Year resolution pursuit, we predicted that goal flexibility and goal tenacity, at baseline, would each significantly and independently predict sustained New Year resolution “stickability”. Finally, we predicted that the interaction between these constructs would be associated with sustained New Year resolution pursuit over time.

## 2. Materials and Methods

### 2.1. Design

To test our hypotheses an online longitudinal study was conducted over a two-month period. All participants commenced the study at or after the New Year but within the first month of the study (January). Baseline measures administered at Time 1 (T1) were followed by three subsequent surveys, with a two-week interval between T1 and Time 2 (T2), and T2 and Time 3 (T3). Literature suggests that most people abandon their New Year resolutions within the first month [3]. Therefore, we included a 4-week interval between T3 (end of the first month) and Time 4 (T4) to ascertain whether those participants still pursuing their most important resolution at T3 were doing so at the end of the following month (T4, end of the second month). A priori sample size calculations were conducted to determine the minimum sample size required for hierarchical regression-based analyses. For a desired statistical power of 0.8, an anticipated medium effect size for model fit and probability level set at 0.05, a minimum sample size of 84 is required. Given an anticipated dropout rate of 50% from Time 1 to Time 4, a minimum sample of 168 was required at Time 1.

### 2.2. Participants

The total sample comprised 182 participants recruited from community and university samples in Australia (*n* = 144) and the United Kingdom (UK; *n* = 38). There were 147 females (80.8%), 31 males (17.0%), 3 participants reported “other” (1.6%) and 1 participant did not report their gender (0.5%). The proportion of females and males did not differ significantly across the four time points (*p’s* > 0.05). However, the proportion of males and females was significantly different across the Australian and UK samples (*χ*^2^(1) = 4.72, *p* = 0.030), with a higher percentage of females in the Australian sample (86.2%) than in the UK sample (68.4%). Ages ranged from 18 to 77 years for the total sample, with a mean age of 37.12 years (*SD* = 14.50). There was a significant difference in mean age (*t*(177) = 3.53, *p* < 0.001), with Australian participants (M*_age_* = 35.20; *SD* = 13.79) being younger than their UK counterparts (M*_age_* = 44.26; *SD* = 15.00).

One hundred and sixty-one participants completed T1, 92 participants completed T2, 65 participants completed T3 and 54 participants completed T4.

### 2.3. Measures

#### 2.3.1. New Year Resolution Task

New Year Resolution Task (adapted from Dickson and Moberly, 2010) [16]: to elicit subjectively meaningful New Year resolutions at T1, participants were asked to think about their New Year resolutions, and to select the “one” resolution to which they were most committed. Participants were instructed that this resolution should be specific enough that they would know whether they were successfully sticking to their resolution over the coming weeks. Participants reported a short single written statement to list their most meaningful New Year resolution to which they were most committed.

#### 2.3.2. Tenacious Goal Pursuit and Flexible Goal Attainment Scale (TEN/FLEX) 

TEN/FLEX [17]: this measure comprises two subscales and was administered at T1 to assess tenacious goal pursuit (TGP; e.g., “When faced with obstacles, I usually double my efforts”) and flexible goal adjustment (FGA; e.g., “In general, I am not upset very long about a missed opportunity”). The TGP comprises 15 items (with 9 reverse-scored items) and the FGA comprises 15 items (with 4 reverse-scored items). Participants rate each item on a scale ranging from −2 (“strongly disagree”) to +2 (“strongly agree”), but when scoring, items are scored from 1 to 5 respectively. TGP and FGA subscale scores can range from 15 to 75, with higher scores indicating greater goal tenacity and higher goal flexibility, respectively. Cronbach’s alphas in the original development paper were 0.80 (TGP) and 0.83 (FGA). The present study showed acceptable and comparable reliabilities, with Cronbach’s α = 0.83 for the TGP and α = 0.80 for the FGA.

#### 2.3.3. Warwick–Edinburgh Mental Wellbeing Scale (WEMWS)

Warwick–Edinburgh Mental Wellbeing Scale (WEMWS) [18]: this measure was administered at each time point (T1-T4) and comprises 14-items to assess mental wellbeing (e.g., “I’ve been feeling interested in other people”). Item statements are rated on a scale ranging from 1 (“none of the time”) to 5 (“all of the time”). Total scores can range from 14 to 70, where higher scores indicate greater wellbeing. The measure has been extensively validated, with high test–retest reliability and an acceptable Cronbach’s alpha (α = 0.91) in a population sample. In the present study the dependent variable WEMWS showed acceptable and comparable reliabilities ranging from α = 0.93 to α = 0.95 across the four time points.

#### 2.3.4. Longitudinal Goal Ratings

In addition to the WEMWS measure, participants rated three items applied to their represented New Year resolution at each time point from T2 through to T4. These included, item 1, commitment (i.e., “To what extent are you still committed to this resolution?”), item 2, stickability (i.e., “In the last two weeks how successful have you been in sticking to this resolution?”), item 3, effort (“i.e., In the last two weeks, to what extent have you put effort into sticking to this resolution?”). Each item was rated on a 7-point scale ranging from 1 (“not at all”) to 7 (“extremely”). Based on the significant positive correlations and large effects (ranging from *r* = 0.58 to 0.73) between the three New Year resolution goal items we combined the commitment, effort and stickability items to form one variable, “Goal Stickability”. Cronbach’s alpha for this aggregated variable was 0.86 at T2, 0.87 at T3 and 0.88 at T4.

We also included an additional item (Item 1a) to ascertain whether participants had abandoned their resolution (i.e., “If you have abandoned this resolution, please provide a brief reason why here (open text box)”). Eight participants reported that they had abandoned their New Year resolution at T2, six participants had abandoned their resolution at T3 and seven participants at T4. The most reported reason for abandoning the New Year resolution was a change in priorities or circumstances (e.g., due to have a baby).

### 2.4. Procedure

Edith Cowan University and Exeter University Psychology Research Ethics Committees approved the study. Following ethics approval, the longitudinal study was administered online via a Qualtrics survey at four time points. All participants provided informed consent before participating in the study. At T1 participants completed the demographic items, listed the New Year resolution to which they were most committed and completed associated items (identified in the following paragraph), and the self-report goal flexibility and goal tenacity, and wellbeing questionnaires. In the follow up surveys (T2, T3 and T4) participants completed the longitudinal New Year resolution items (commitment, effort and stickability) and the wellbeing measure (WEMWS).

At baseline (T1) we included items to check whether participants had decided on their New Year resolution prior to the survey: (i) “had you decided on this New Year resolution before beginning this survey?” and whether they had the same resolution in previous years (ii) “have you had this New Year resolution in previous years?”. Most participants reported that they had decided on the listed New Year resolution prior to commencing the survey (*n* = 134; 73.2%), while almost a quarter of the sample (*n* = 43, 23.5%) reported that their resolution was “partially” decided prior to the survey, and only five participants (2.7%) reported not having decided upon their listed resolution prior to the survey. Nearly a quarter of participants reported they had this same resolution in previous years (*n* = 44, 24.0%), nearly a third of participants (*n* = 53, 29.0%) reported they partially had the same resolution in previous years, while almost half of the participants had selected a new resolution (*n* = 85, 46.4%), with one participant not responding (0.5%). We also checked participants’ assessments of their “commitment” and “importance” of their resolution at baseline (T1). Based on 7-point scales from 1 (“not at all”) to 7 (“extremely”), participants were strongly committed to their resolution (*M* = 5.94, *SD* = 0.97) and considered it very important to stick to their resolution (*M* = 6.03, *SD* = 1.05). In summary, at baseline, most participants had previously set, or partially set, the same resolution in previous years and had decided on their resolution prior to taking part in the study. Participants reported high commitment to the resolution listed and rated highly the importance of sticking to the listed resolution. Participants also reported that it was unlikely that they would abandon their New Year resolution when faced with a setback (*M* = 2.29, *SD* = 1.44), which was rated on a 7-point scale from 1 (“not at all”) to 7 (“extremely”).

We coded each listed New Year resolution for (i) orientation, (ii) specificity and (iii) content. Each resolution was coded as either approach or avoidance oriented. An approach resolution is focused on a desired outcome and involves striving toward the positive outcome (e.g., “I will eat healthily”), whereas an avoidance resolution is focused on an undesired outcome and involves trying to prevent or inhibit the negative outcome (e.g., “I will avoid fatty foods”). Most participants listed resolutions that were approach oriented (88.7%), whereas only 11.3% listed resolutions that were avoidance oriented. These motivational orientation findings are consistent with previous research using community and university samples [16]. Although, this pattern was further amplified for those participants who completed all four time points, with 96% of completers having listed approach resolutions, relative to only 4% of completers having listed avoidance oriented resolutions. Inter-rater reliability yielded good reliability for the approach and avoidance resolutions (*K* = 1).

A dichotomous coding scheme was used to categorise New Year resolution specificity [19]. A New Year resolution was described as “specific” if it described an explicit aim or target feature and included at least one of the following specific aspects: time, place or people (e.g., “to go for a 40-minute walk around the lake four times a week”). A New Year resolution was described as “general” if it referred to a global or abstract resolution (e.g., “to get fit”). At baseline, most New Year resolutions (64.6%) were described in very broad or general terms and approximately one third were specific (35.4%). The coding scheme yielded good inter-rater reliability (*K* = 1). New Year resolutions were also categorised according to one of the following content domains: exercise, diet, academic or skill development, mental wellbeing, bad habits, money or finances, leisure activity, interpersonal relationships, work or occupation, a “cause” (e.g., environmental causes) and time management. Over 50% of the resolutions listed focused on diet (29.0%), for example, “Aim to lose 5 kg” and exercise (24.6%), for example, “30+ mins exercise 5 × per week”. Inter-rater reliability was good (*K* = 1).

## 3. Results

No significant differences were found between the Australian and UK samples on any of the main study variables (all *p’s* > 0.05). Therefore, subsequent regression analyses were conducted on the total sample at each time point to test the hypotheses.

Descriptive statistics and Pearson’s *r* correlations between the main study variables are shown in Table 1.

As can be seen in Table 1, flexible goal adjustment was significantly and positively associated with mental wellbeing (WEMWS) at each time point, showing medium to large effects (*r*’s ranging from 0.40 to 0.52) but had no significant relationship with New Year resolution “stickability” across time (*p*’s > 0.05). Tenacious goal pursuit correlated significantly with wellbeing at T1 (*r* = 0.30), but counter to prediction did not correlate with wellbeing at any other time point. Nor did tenacious goal pursuit correlate significantly with New Year resolution “stickability” across time (*p*’s > 0.05). As can be seen in Table 1, and as expected, wellbeing assessments were significantly correlated at each time point (ranging from *r* = 0.55 to 0.78), as were New Year resolution “stickability” assessments (ranging from *r* = 0.54 to 0.72). Age did not correlate significantly with any of the main study variables (all *p*’s > 0.05). No significant gender differences were found on the main study variables, except for flexible goal adjustment (*t*(155) = 2.09, *p* = 0.039), where males (*M* = 38.94, *SD* = 7.42) reported greater goal flexibility than females (*M* = 35.19, *SD* = 9.58).

Prior to conducting our main analyses, dropout analyses were conducted, based upon baseline assessments at Time 1, comparing participants who completed all phases of the study against non-completers (see Table S1 in Supplementary Materials). There were no significant differences between study completers and non-completers on baseline (Time 1) goal tenacity (TGP), flexible goal adjustment (FGA), mental wellbeing (WEMH) or the proportion of males and females in each sample, suggesting that our predictors were not confounded with dropout from the study. However, study completers were significantly older than non-completers (*M_Completers_* = 43.10, *SD* = 15.46 vs. *M_Non-completers_* = 34.98, *SD* = 13.58; *t*(176) = −3.41, *p* = 0.001).

Given that some participants reactivated or partially reactivated previous resolutions, whilst others selected new resolutions, we also examined if there were any differences between these groups on the main study variables at baseline. No significant differences were found between these groups on TGP, FGA, WEMWS nor on age or gender (all *p*’s > 0.05).

Unfortunately, sample sizes obtained at each time point precluded the use of more advanced longitudinal analyses, such as latent growth curve modelling. More pertinently, the aim of this study was not the examination of change over time (as in latent growth modelling) but examining predictors of mental wellbeing and New Year resolution goal stickability at different time intervals. Therefore, to test our main hypotheses, we conducted hierarchical regression analyses to investigate if tenacious and flexible goal pursuit assessed at baseline (T1) each independently predicted wellbeing and New Year resolution “stickability”, respectively, across time, while controlling for participant gender. The regression analyses also tested whether the interaction between goal tenacity and flexibility made an additional contribution to wellbeing and sustained New Year resolution pursuit (i.e., goal stickability). In the regression model, gender, TGP and FGA were entered on Step 1, and the interaction term between TGP and FGA was entered on Step 2. Regression results are presented in Table 2 and Table 3 below. To control for multiple tests at each time point, we applied a Bonferroni correction, with the predictor variables considered significant predictors if probability falls below 0.0125 (i.e., 0.05/4) when predicting wellbeing (WEMWS) across four time points, and below 0.0167 (i.e., 0.05/3) when predicting goal stickability across three time points.

As shown in Table 2, and as predicted, flexible goal adjustment independently, and significantly predicted wellbeing at Times 1–3, and approached significance at Time 4 (*p* = 0.017). Counter to prediction, tenacious goal pursuit did not predict wellbeing at any time point. As shown in Table 3, FGA predicted goal stickability at Time 2, but not at Time 3 and 4. Counter to prediction, TGP did not predict sustained New Year resolution goal stickability at any time point, nor did the interaction between goal flexibility and tenacity significantly predict wellbeing or New Year resolution stickability.

Additional supplementary regression analyses were conducted based upon the model discussed above (i.e., gender, TGP, FGA and the interaction term TGP × FGA were included on the same steps) to examine if baseline “commitment” and “stickability importance” of New Year’s resolutions predicted wellbeing and goal stickability over time, respectively (see Supplementary Materials, Tables S2 and S3). Based on Bonferroni corrections, neither baseline commitment nor New Year resolution importance predicted wellbeing at any time point. Commitment predicted New Year resolution stickability at Time 3 but not at Time 2 or 4, while importance did not predict stickability at any time point. Next, we examined if the New Year resolution orientation (i.e., approach vs. avoidance) and specificity (i.e., specific vs. general) predicted wellbeing and sticking to one’s resolution over time. Neither resolution orientation nor specificity predicted wellbeing or stickability at any time point (see Supplementary Materials, Tables S4 and S5).

## 4. Discussion

We aimed to investigate whether goal flexibility, goal tenacity and their interaction at baseline each independently predicted greater wellbeing and sustained New Year resolution pursuit. As expected, preliminary correlations showed that wellbeing assessments were positively correlated across time, as were resolution “stickability” assessments. As predicted, goal flexibility at baseline predicted wellbeing over time but counter to prediction it did not predict sticking to one’s New Year resolution. Counter to prediction, goal tenacity, at baseline, predicted neither wellbeing nor sustained New Year resolution pursuit over time. Nor did the interaction between flexibility and tenacity significantly predict enhanced wellbeing or resolution stickability. Participants’ self-generated New Year resolutions were mostly approach oriented, tended to focus on “diet” and “exercise”, had been set at least partially in previous years, and tended to be described in very general terms.

### 4.1. Goal Flexibility

The fact that goal flexibility predicted wellbeing across time supports our hypothesis and is consistent with past research [13,14]. The findings lend support to the view that flexible goal adjustment and one’s capacity to view difficulties with composure and a degree of detachment augments the ability to adapt one’s approach to life situations when required, which in turn promotes wellbeing [11]. Enhanced goal flexibility promotes wellbeing as it allows people to meet personal and societal expectations by adjusting to life’s ever-changing constraints and opportunities, and to feel more autonomous in relation to the self and the future [20].

Although New Year resolutions represent a form of personal goal setting, they may differ from conventional goals because they are more often set and pursued in a social context, given the cultural emphasis on New Year resolutions in Western cultures. Hence, they may be more externally regulated and more difficult to pursue than other goals. Research indicates that more internally regulated goals are associated with increases in positive wellbeing, and as such are more likely to assist sustained goal pursuit [21,22]. However, without direct measures to assess the underlying reasons for New Year resolution pursuit, the role of external versus internal regulation in New Year resolution pursuit remains unclear. It would be useful for future research to address this issue.

Further, the TEN/FLEX flexible goal adjustment measure did not differentiate between people’s ability, when resolution pursuit is difficult, to choose alternative means to fulfil the resolution or to abandon the resolution altogether in favour of more fruitful pursuits. These tendencies may pull in opposite directions in terms of the stickability of New Year resolutions, such that their net association is near negligible.

### 4.2. Goal Tenacity

In contrast to previous research [13,14], and counter to prediction, goal tenacity at baseline did not independently predict greater wellbeing throughout the study. Nor did it predict sustained New Year resolution pursuit. Developmental differences might partly explain the present non-significant findings, as past research has predominantly focused on older adults [13,14]. For instance, older individuals may rely more on personal tenacity to maintain their sense of wellbeing, relative to younger individuals. Participants in this study were predominantly middle-aged. It may be that people within this age group have more competing goals and demands, and fewer resources (e.g., time and money) to stick to their New Year resolutions. The lack of significant findings may also relate to the measure itself. While goal tenacity has been defined by “persistence”, it could be argued there is a fine line between “persistent” tenacity and “rigid” tenacity. For instance, it is possible that tenacity taps into a form of goal inflexibility. If so, this may in part account for the non-significant findings in relation to tenacity and wellbeing, and tenacity and the sustained pursuit of New Year resolutions. Arguably, rigidity and inflexibility are likely to make it more difficult to adjust or adapt a resolution in the face of difficulty or in response to lack of progress, or it could lead to “all-or-nothing” thinking such that a goal is abandoned rather than adjusted. Past research also suggests that goal tenacity may be associated with psychopathology and perfectionism, particularly if someone continues to pursue goals that are unrealistic and unattainable [21]. Further, several studies have associated goal inflexibility with a range of psychological disorders (e.g., depression and anxiety) and maladaptive psychological mechanisms such as negative rumination [5,23,24].

### 4.3. New Year Resolution Characteristics

The coding categorisation for content found that more than half the sample listed either a “diet” or “exercise” New Year resolution and just over half the sample reported having had the same or a similar resolution in the past year. Together these findings suggest that people’s New Year resolutions often reflect cyclical goals that are “rebooted” each New Year. Speculatively, this circularity may indicate that people choose past resolutions because they are difficult, and hence fail to stick to them in the current year. Further, most participants in the present study listed very general or abstract resolutions, which arguably renders sustained New Year resolution pursuit more difficult. Setting specific New Year resolutions is more likely to suggest the necessary strategies and plans to aid sustained and successful pursuit and to promote wellbeing, relative to abstract or vague resolutions [19].

Goals are hierarchically organised with abstract superordinate resolutions representing what people ultimately value and aspire to [2,25,26,27]. New Year resolutions are also likely to be hierarchically organised and interconnected with other goals and, consequently, may facilitate or hinder other pursuits [28]. Future research could usefully examine whether linking people’s more abstract resolutions to more concrete lower order resolutions facilitate sustained New Year resolution pursuit and enhanced wellbeing over time. Except for a recent article published by Höchli and colleagues in 2020 [29], little research has investigated how superordinate and subordinate goals influence and interact with each other to support New Year resolution pursuit. For example, if the New Year resolution “to lose 5 kg in weight” is consistent with superordinate goals, such as beliefs about one’s personal health or appearance, or vice versa, then this is likely to aid sustained motivation. Future research could examine whether explicitly linking superordinate and subordinate goals and plans improves New Year resolution sustainability and wellbeing.

### 4.4. Methodological Considerations

A few methodological considerations deserve comment. There was a sizable attrition rate from baseline to the final survey (T4). This attrition rate is consistent with past literature, which indicates that people typically “give up” pursuing their New Year resolutions within the first month [3]. However, in the present study there were no significant differences between study completers and non-completers on the key baseline measures (goal tenacity, goal flexibility and mental wellbeing). Thus, the central variables investigated in this study do not seem to be confounded with successful completion of the study, which suggests that it is possible to generalise the study results despite the high attrition rate. The sample size was not sufficient to use multilevel modelling to investigate different resolutions within individuals to examine characteristics of resolutions that predict stickability within persons, and may have led to reduced power to detect main effects and interactions. Future research using larger samples could usefully include more covariates to differentiate goal flexibility and goal tenacity from neighbouring constructs (e.g., optimism, neuroticism and grit). The supplementary analyses did not change the significant and non-significant results reported in the main text. Although including the covariates “commitment” and “importance” at baseline did not change the significant and non-significant findings over time, these supplementary analyses were underpowered due to the small sample sizes. Future research may benefit from the use of incentives (e.g., prize draws) to increase potential sample sizes. The self-report nature of the study is a limitation and social desirability may have inflated associations between goal flexibility and wellbeing, but the null findings between goal tenacity and wellbeing tend to counter this possibility. Although we asked participants to rate the subjective stickability of their resolutions, future research could usefully investigate more objective measures of success in resolution pursuit.

Overall, our results tend to support the view that people are not particularly good at sticking to their New Year resolutions. This is despite participants initially reporting high importance of and commitment to the resolution and the belief that they would stick to their resolution even in the face of obstacles and difficulties. Although our results showed positive associations between goal flexibility and wellbeing, there was no direct relationship between goal flexibility and sticking to one’s New Year resolution. Given the high proportion of resolutions that were reported to be the same, or almost the same, as those set in a previous year, it appears that people readily disengage behaviourally from pursuing their New Year resolutions but may not necessarily disengage cognitively. This might, in part, explain why the same resolutions are readily reactivated each New Year. These assumptions await further investigation.

## 5. Conclusions

Overall, the results indicate that trait levels of neither goal flexibility nor tenacity support sustained pursuit of New Year resolutions. However, flexible goal pursuit (but not tenacious goal pursuit) is associated with higher levels of mental wellbeing. Although setting New Year resolutions is a popular activity in many cultures, our findings are consistent with the folk wisdom that people are not particularly good at sticking to them. Future research, using larger samples may benefit by focusing on the predictive role of alternative trait characteristics on resolution stickability, in addition to goal-level characteristics such as the extent to which resolutions are pursued for internalized reasons.

## Figures and Tables

**Table 1:** Descriptive statistics and Pearson’s r correlation between study variables.

Variable	2.	3.	4.	5.	6.	7.	8.	9.	Mean (*SD*)
1. TGP	0.36 ***	0.30 ***	0.17	0.07	0.22	0.13	0.13	0.00	36.10 (10.27)
2. FGA	-	0.52 ***	0.43 ***	0.48 ***	0.40 **	0.10	0.10	0.19	35.90 (9.30)
3. T1 WEMWS		-	0.60 ***	0.62 ***	0.76 ***	0.07	0.07	0.06	48.89 (9.81)
4. T2 WEMWS			-	0.74 ***	0.55 ***	0.29 *	0.29 *	0.28 *	47.52 (9.13)
5. T3 WEMWS				-	0.78 ***	0.24	0.24	0.31 *	48.55 (8.24)
6. T4 WEMWS					-	0.21	0.21	0.31 *	48.77 (9.07)
7. T2 Stickability						-	0.70 ***	0.54 ***	14.80 (4.27)
8. T3 Stickability							-	0.72 ***	13.92 (4.87)
9. T4 Stickability								-	14.46 (4.55)

Note: * = *p* < 0.05, ** = *p* < 0.01, *** = *p* < 0.001. T = Time; TGP = Tenacious Goal Pursuit; FGA = Flexible Goal Adjustment; WEMWS = Warwick–Edinburgh Mental Wellbeing Scale.

**Table 2:** Tenacious goal pursuit and flexible goal adjustment as predictors of T1 to T4 wellbeing.

	T1 WEMWS (*n* = 156)					T2 WEMWS (*n* = 88)					T3 WEMWS (*n* = 64)					T4 WEMWS (*n* = 53)				
Variable	*β*	*b* (SE)	95% CIs	*t*	*p*	*β*	*b* (SE)	95% CIs	*t*	*p*	*β*	*b* (SE)	95% CIs	*t*	*p*	*β*	*b* (SE)	95% CIs	*t*	*p*
Gender	0.04	1.04 (1.69)	−2.30, 4.38	0.61	0.541	−0.04	−0.93 (2.41)	−5.72, 3.86	−0.39	0.701	−0.04	0.88 (2.66)	−4.45, 6.21	0.33	0.743	0.06	1.42 (3.25)	−5.11, 7.95	0.44	0.663
T1 TGP	0.13	0.11 (0.07)	−0.02, 0.25	1.69	0.094	0.07	0.07 (0.10)	−0.13, 0.27	0.69	0.495	−0.01	−0.01 (0.10)	−0.21, 0.20	−0.09	0.929	0.14	0.14 (0.13)	−0.12, 0.40	1.09	0.283
T1 FGA	0.47	0.47 (0.08)	0.32, 0.62	6.16	<0.001	0.44	0.44 (0.11)	0.23, 0.65	4.14	<0.001	0.48	0.47 (0.11)	0.25, 0.70	4.17	<0.001	0.33	0.39 (0.16)	0.07, 0.71	2.46	0.017
T1 TGP × T1 FGA	−0.04	0.00 (0.01)	−0.02, 0.01	−0.62	0.540	0.15	0.01 (0.01)	−0.01, 0.03	1.37	0.174	0.16	0.02 (0.01)	−0.01, 0.04	1.43	0.158	0.17	0.02 (0.02)	−0.01, 0.06	1.30	0.200
Model:	*R*^2^ = 0.29, *F*(4,151) = 15.43 ***					*R*^2^ = 0.21, *F*(4,83) = 5.35 **					*R*^2^ = 0.26, *F*(4,59) = 5.06 **					*R*^2^ = 0.21, *F*(4,48) = 3.14 *				

Note. * = *p* < 0.05, ** = *p* < 0.01, *** *p* < 0.001; T = time; TGP = Tenacious Goal Pursuit; FGA = Flexible Goal Adjustment; WEMHS = Warwick–Edinburgh Mental Wellbeing Scale.

**Table 3:** Tenacious goal pursuit and flexible goal adjustment as predictors of T2 to T4 goal stickability.

	T2 Stickability (*n* = 91)					T3 Stickability (*n* = 63)					T4 Stickability (*n* = 54)				
Variable	*β*	*b* (SE)	95% CIs	*t*	*p*	*β*	*b* (SE)	95% CIs	*t*	*p*	*β*	*b* (SE)	95% CIs	*t*	*p*
Gender	0.04	0.43 (1.12)	−1.80, 2.66	0.38	0.703	0.00	0.05 (1.81)	−3.57, 3.67	0.03	0.978	0.05	0.58 (1.77)	−2.98, 4.14	0.33	0.746
T1 TGP	0.14	0.06 (0.05)	−0.03, 0.15	1.35	0.181	0.11	0.06 (0.07)	−0.08, 0.20	0.82	0.413	−0.03	−0.02 (0.07)	−0.16, 0.12	−0.23	0.818
T1 FGA	0.32	0.15 (0.05)	0.05, 0.24	3.02	0.003	0.08	0.05 (0.08)	−0.11, 0.20	0.60	0.550	0.18	0.11 (0.09)	−0.07, 0.28	1.24	0.221
T1 TGP × T1 FGA	−0.12	−0.01 (0.00)	−0.01, 0.00	−1.17	0.244	−0.05	0.00 (0.01)	−0.02, 0.01	−0.41	0.684	0.12	0.01 (0.01)	−0.01, 0.02	0.81	0.422
Model:	*R*^2^ = 0.20, *F*(4,86) = 5.43 **					*R*^2^ = 0.02, *F*(4,58) = 0.36					*R*^2^ = 0.05, *F*(4,49) = 0.70				

Note: ** = *p* < 0.01; T = time; TGP = Tenacious Goal Pursuit; FGA = Flexible Goal Adjustment; WEMHS = Warwick–Edinburgh Mental Wellbeing Scale.

## Data Availability

Anonymous data was collected and save on an SPSS datafile. This SPSS dataset will be deposited at Edith Cowan University’s data repository and accession numbers will be made publically available prior to publication of the article.

## Supplementary Materials

The following are available online at https://www.mdpi.com/1660-4601/18/6/3084/s1, Table S1: Dropout analyses, comparing study completers (all phases) versus non-completers on baseline TGP, FGA, WEMWS, Table S2: Tenacious Goal Pursuit, Flexible Goal Adjustment, Resolution Commitment and Stickability Importance as predictors of T1 to T4 wellbeing, Table S3: Tenacious Goal Pursuit, Flexible Goal Adjustment, Resolution Commitment and Importance as predictors of T2 to T4 stickability, Table S4: Tenacious Goal Pursuit, Flexible Goal Adjustment, and Resolution Orientation (approach vs. avoid) and Specificity (specific vs. general) as predictors of T1 to T4 wellbeing, Table S5: Tenacious Goal Pursuit, Flexible Goal Adjustment, and Resolution Orientation (approach vs. avoid) and Specificity (specific vs. general) as predictors of T2 to T4 stickability.
