# Supplementary material for: Self-Regulatory Goal Motivational Processes in Sustained New Year Resolution Pursuit and Mental Wellbeing
Source: Int J Environ Res Public Health. 2021 Mar 17;18(6):3084. doi: 10.3390/ijerph18063084 (PMC8002459; doi:10.3390/ijerph18063084)
Supplement: Supplementary file 1 [file ijerph-18-03084-s001.pdf]

Table S1. Dropout analyses, comparing study completers (all phases) versus non-completers on baseline TGP, FGA, WEMWS.

Variable	Completers (n = 48) *M (SD)*	Non-completers (n = 116) *M (SD)*	Independent samples t-tests
T1 TGP	36.27 (9.17)	35.90 (10.68)	$t(162) = -0.21$, $p = .832$
T1 FGA	34.17 (7.69)	36.60 (9.88)	$t(160) = 1.52$, $p = .130$
T1 WEMWS	49.77 (7.83)	48.44 (10.57)	$t(158) = -0.79$, $p = .433$

Note. Missing data for non-completers at: T1 FGA (n = 2), T1 WEMWS (n = 4). T1 = Time 1; TGP =Tenacious Goal Pursuit; FGA = Flexible Goal Adjustment; WEMWS = Warwick-Edinburgh Mental Wellbeing Scale.

Table S2. Tenacious Goal Pursuit, Flexible Goal Adjustment, Resolution Commitment and Stickability Importance as predictors of T1 to T4 wellbeing.

**Variable**	**T1 WEMWS (*n* = 156)**					**T2 WEMWS (*n* = 88)**					**T3 WEMWS (*n* = 64)**					**T4 WEMWS (*n* = 53)**				
	β	***b* (SE)**	**95% CIs**	***t***	***p***	***β***	***b* (SE)**	**95% CIs**	***t***	***p***	***β***	***b* (SE)**	**95% CIs**	***t***	***p***	***β***	***b* (SE)**	**95% CIs**	***t***	***p***
Gender	.04	1.04 (1.71)	-2.35, 4.43	0.61	.544	-.05	-1.20 (2.46)	-6.10, 3.70	-0.49	.627	.02	0.37 (2.67)	-4.99, 5.72	0.14	.892	.01	0.21 (3.43)	-6.70, 7.12	0.06	.952
Commitment	-.02	-0.23 (0.93)	-2.07, 1.61	-0.26	.807	.09	0.94 (1.42)	-1.89, 3.76	0.66	.510	.22	2.02 (1.34)	-0.65, 4.70	1.52	.135	.18	1.85 (1.89)	-1.95, 5.64	0.98	.333
Importance	.08	0.76 (0.80)	-0.83, 2.34	0.94	.348	-.11	-1.20 (1.44)	-4.06, 1.66	-0.84	.405	-.08	-0.82 (1.46)	-3.74, 2.10	-0.56	.577	.00	0.02 (1.91)	-3.82, 3.85	0.01	.994
T1 TGP	.12	0.10 (0.07)	-0.03, 0.24	0.94	.133	.08	0.08 (0.10)	-0.12, 0.28	0.77	.445	-.02	-0.02 (0.10)	-0.23, 0.19	-0.20	.846	.14	0.13 (0.13)	-0.13, 0.39	1.01	.319
T1 FGA	.47	0.47 (0.08)	0.32, 0.62	6.04	< .001	.43	0.43 (0.11)	0.21, 0.64	3.94	< .001	.46	0.45 (0.11)	0.22, 0.68	3.98	< .001	.30	0.35 (0.16)	0.03, 0.68	2.16	.036
T1 TGP x T1 FGA	-.04	0.00 (0.01)	-0.01, 0.01	-0.61	.542	.16	0.01 (0.01)	-0.01, 0.03	1.46	.148	.19	0.02 (0.01)	0.00, 0.05	1.66	.103	.21	0.03 (0.02)	-0.01, 0.06	1.53	.133
Model:	R^2 = .30, F(6,149) = 10.38***					R^2 = .21, F(6,81) = 3.64**					R^2 = .29, F(6,57) = 3.81**					R^2 = .24, F(6,46) = 2.38*				

Note. * = p < .05, ** = p < .01, *** p < .001; T = time; TGP = Tenacious Goal Pursuit; FGA = Flexible Goal Adjustment; WEMHS = Warwick-Edinburgh Mental Wellbeing Scale.

Table S3. Tenacious Goal Pursuit, Flexible Goal Adjustment, Resolution Commitment and Importance as predictors of T2 to T4 stickability.

Variable	T2 Stickability (*n* = 91)					T3 Stickability (*n* = 63)					T4 Stickability (*n* = 54)				
	**β**	***b* (SE)**	**95% CIs**	***t***	***p***	**β**	***b* (SE)**	**95% CIs**	***t***	***p***	**β**	***b* (SE)**	**95% CIs**	***t***	***p***
Gender	.00	0.04 (1.09)	-2.13, 2.21	0.03	.973	-.04	-0.60 (1.71)	-4.02, 2.83	-0.35	.729	-.04	-0.44 (1.82)	-4.10, 3.22	-0.24	.810
Commitment	.24	1.23 (0.62)	-0.01, 2.47	1.98	.051	.46	2.53 (0.86)	0.81, 4.24	2.95	.005	.30	1.54 (1.00)	-0.48, 3.56	1.54	.131
Importance	.06	0.34 (0.63)	-0.91, 1.59	0.54	.593	-.14	-0.87 (0.98)	-2.84, 1.10	-0.89	.380	.01	0.07 (1.01)	-1.97, 2.11	0.07	.947
T1 TGP	.14	0.06 (0.04)	-0.03, 0.15	1.39	.169	.08	0.04 (0.07)	-0.09, 0.17	0.64	.526	-.05	-0.02 (0.07)	-0.16, 0.11	-0.35	.729
T1 FGA	.27	0.12 (0.05)	0.03, 0.22	2.63	.010	.04	0.02 (0.07)	-0.13, 0.17	0.28	.780	.13	0.08 (0.09)	-0.10, 0.25	0.90	.373
T1 TGP x T1 FGA	-.10	0.00 (0.00)	-0.01, 0.00	-1.00	.320	.01	0.00 (0.01)	-0.02, 0.02	0.06	.952	.17	0.01 (0.01)	-0.01, 0.03	1.22	.228
Model:	R^2 = .28, $F(6,84)$ = 5.44***					R^2 = .17, $F(6, 56)$ = 1.92					R^2 = .14, $F(6,47)$ = 1.27				

Note. *** $p < .001$; T = time; TGP = Tenacious Goal Pursuit; FGA = Flexible Goal Adjustment; WEMHS = Warwick-Edinburgh Mental Wellbeing Scale.

Table S4. Tenacious Goal Pursuit, Flexible Goal Adjustment, and Resolution Orientation (approach vs. avoid) and Specificity (specific vs. general) as predictors of T1 to T4 wellbeing.

	**T1 WEMWS (*n* = 155)**					**T2 WEMWS (*n* = 87)**					**T3 WEMWS (*n* = 63)**					**T4 WEMWS (*n* = 52)**				
**Variable**	***β***	***b* (SE)**	**95% CIs**	***t***	***p***	***β***	***b* (SE)**	**95% CIs**	***t***	***p***	***β***	***b* (SE)**	**95% CIs**	***t***	***p***	***β***	***b* (SE)**	**95% CIs**	***t***	***p***
Gender	.02	0.53 (1.74)	-2.92, 3.97	0.30	.764	-.02	-0.54 (2.47)	-5.17, 8.30	-0.22	.827	.03	0.66 (2.73)	-4.81, 6.13	0.24	.809	.07	1.69 (3.32)	-5.01, 8.38	0.51	.614
Orientation	-.08	-2.36 (2.11)	-6.54, 1.82	-1.12	.266	.05	1.57 (3.89)	-5.17, 8.30	0.46	.645	-.11	-2.78 (3.29)	-9.37, 3.82	-0.84	.403	-.06	-2.85 (6.61)	-16.14, 10.45	-0.43	.668
Specificity	-.07	-1.34 (1.40)	-4.10, 1.43	-0.96	.340	.09	1.77 (2.00)	-2.22, 5.76	0.89	.379	.02	0.35 (2.06)	-3.78, 4.48	0.17	.866	-.22	-4.40 (2.61)	-9.65, 0.85	-1.69	.098
T1 TGP	.13	0.12 (0.07)	-0.01, 0.26	1.78	.077	.09	0.09 (0.10)	-0.12, 0.29	0.85	.396	.01	0.01 (0.11)	-0.20, 0.22	0.06	.953	.16	0.15 (0.14)	-0.12, 0.43	1.12	.267
T1 FGA	.48	0.48 (0.08)	0.33, 0.63	6.25	< .001	.43	0.43 (0.11)	0.21, 0.64	3.96	< .001	.50	0.49 (0.12)	0.26, 0.73	4.22	< .001	.33	0.38 (0.16)	0.06, 0.70	2.40	.020
T1 TGP x T1 FGA	-.05	0.00 (0.01)	-0.02, 0.01	-0.65	.516	.16	0.01 (0.01)	-0.01, 0.03	1.48	.143	.15	0.02 (0.01)	-0.01, 0.04	1.22	.227	.16	0.02 (0.02)	-0.02, 0.06	1.17	.247
Model:	R^2 = .30, F(6,148) = 10.63***					R^2 = .22, F(6,80) = 3.73**					R^2 = .27, F(6,56) = 3.46**					R^2 = .26, F(6,45) = 2.62*				

Note. * = $p < .05$, ** = $p < .01$, *** $p < .001$; T = time; TGP = Tenacious Goal Pursuit; FGA = Flexible Goal Adjustment; WEMHS = Warwick-Edinburgh Mental Wellbeing Scale.

Table S5. Tenacious Goal Pursuit, Flexible Goal Adjustment, and Resolution Orientation (approach vs. avoid) and Specificity (specific vs. general) as predictors of T2 to T4 stickability.

	**T2 Stickability (*n* = 90)**					**T3 Stickability (*n* = 62)**					**T4 Stickability (*n* = 53)**				
**Variable**	***β***	***b* (SE)**	**95% CIs**	***t***	***p***	***β***	***b* (SE)**	**95% CIs**	***t***	***p***	***β***	***b* (SE)**	**95% CIs**	***t***	***p***
Gender	.03	0.39 (1.16)	-1.91, 2.69	0.34	.735	-.02	-0.21 (1.84)	-3.90, 3.49	-0.11	.910	.06	0.71 (1.82)	-2.95, 4.37	0.39	.698
Orientation	.02	0.27 (1.58)	-2.87, 3.41	0.17	.864	-.14	-2.11 (2.22)	-3.90, 3.49	-0.95	.346	.06	1.50 (3.63)	-5.80, 8.80	0.41	.681
Specificity	-.04	-0.31 (0.91)	-2.12, 1.50	-0.34	.733	-.05	-0.45 (1.40)	-3.25, 2.34	-0.33	.746	-.15	-1.43 (1.40)	-4.25, 1.39	-1.02	.312
T1 TGP	.14	0.06 (0.05)	-0.04, 0.15	1.24	.218	.11	0.06 (0.07)	-0.08, 0.20	0.81	.421	-.08	-0.04, (0.08)	-0.19, 0.11	-0.48	.632
T1 FGA	.32	0.14 (0.05)	0.05, 0.24	2.91	.005	.09	0.05 (0.08)	-0.11, 0.21	0.66	.514	.15	0.09 (0.09)	-0.09, 0.26	1.02	.313
T1 TGP x T1 FGA	-.13	0.01 (0.00)	-0.01, 0.00	-1.14	.257	-.09	-0.01 (0.01)	-0.02, 0.01	-0.68	.501	.10	0.01 (0.01)	-0.01, 0.03	0.65	.518
Model:	R^2 = .20, F(6,83) = 3.43**					R^2 = .04, F(6, 55) = 0.35					R^2 = .07, F(6,46) = 0.59				

Note. ** = p < .01; T = time; TGP = Tenacious Goal Pursuit; FGA = Flexible Goal Adjustment; WEMHS = Warwick-Edinburgh Mental Wellbeing Scale.
